# Technique for the safe placement of a biodegradable stent into the common bile duct of rabbits

**DOI:** 10.3892/etm.2013.1276

**Published:** 2013-08-28

**Authors:** YIGANG CHEN, JUN YAN, ZHIGANG WANG, SONG YU, ZIMING YUAN, XIAOHU WANG, XIAONONG ZHANG, QI ZHENG

**Affiliations:** 1 Shanghai Jiaotong University School of Medicine, Shanghai 200025;; 2 Department of General Surgery, Sixth People’s Hospital, Shanghai Jiao Tong University School of Medicine, Shanghai 200233;; 3 State Key Laboratory of Metal Matrix Composites, School of Materials Science and Engineering, Shanghai Jiao Tong University, Shanghai 200240, P.R. China

**Keywords:** biodegradable stent, animal experiment, common bile duct

## Abstract

Biodegradable common bile duct (CBD) stents are in high clinical demand. Animal experiments concerning the surgical placement of biliary stents made of new materials are being performed more frequently than ever before. However, these animal experiments only use large animals. In this study, a central venous catheterization set was used as a modified stent introducer system in rabbits. A biodegradable Mg-6Zn alloy CBD stent was passed through the duodenal papilla using this stent introducer system. Computed tomography (CT) scanning of the CBD stent *in vivo* and levels of serum lipase (LPS) were investigated. Twelve rabbits underwent CBD stent insertion and one animal died due to an anesthetic accident. After 3 weeks, when the remaining 11 rabbits were sacrificed, no jaundice or bile leakage was observed. CT scanning of the 11 rabbits suggested that the biodegradable Mg-6Zn stent was successfully placed into the CBD. When the preoperative and postoperative levels of LPS were compared, no statistically significant differences were observed. This new method appears to be feasible and safe for the placement of stents into the CBDs of small animals. This new method can increase the animal number of CBD stent experiment, and improve the quality of experiments.

## Introduction

Diseases of the common bile duct (CBD) represent a significant danger to the patient, since they may lead to obstructive jaundice, biliary colic, cholangitis or pancreatitis (1,2). Traditionally, surgeons explore the CBD and then conduct T-tube drainage following the removal of CBD stones or lesions. It is believed that a T-tube is necessary since it allows spasm or edema of sphincter to settle following the trauma of the exploration. Despite this potential advantage, T-tube-associated complications, including CBD obstruction and bile leakage, often occur after CBD surgery (3–5). An indwelling T-tube requires prescription by a doctor, continuous management and the restriction of patient activity. At the end of treatment, the process of T-tube removal may cause pain and risk to the patient (6–8). The avoidance of complications of the T-tube and the need to support the bile duct are the major challenges faced by surgeons and researchers.

Since biodegradable implant materials in the human body may be gradually dissolved and absorbed, secondary surgery to remove the implants is not required (9). To date, a great number of studies have reported on biodegradable CBD stents in animal experiments (10–12). However, there are certain limitations for the animal experiments, since they are often conducted in large animals such as pigs or dogs (usually with a small sample size of 4–7 dogs or pigs). Large animals have a relatively large CBD which is convenient for the CBD surgery. However, large animals such as dogs and pigs have two disadvantages. Firstly, they are expensive, which limits the number of experimental animals that may be used. Secondly, there are few antibodies against pigs or dogs, which add extra difficulties for molecular experiments in the CBD.

The current study describes a new technique designed for the placement of biodegradable stents into the CBDs of rabbits. The purpose of this study was to provide a safe CBD surgical method in order to enable the sample size of the animals to be expanded and to increase the credibility of experiments concerning biodegradable CBD stents in animals.

## Materials and methods

### Biodegradable CBD stent and stent introducer system

**Figure 1.**
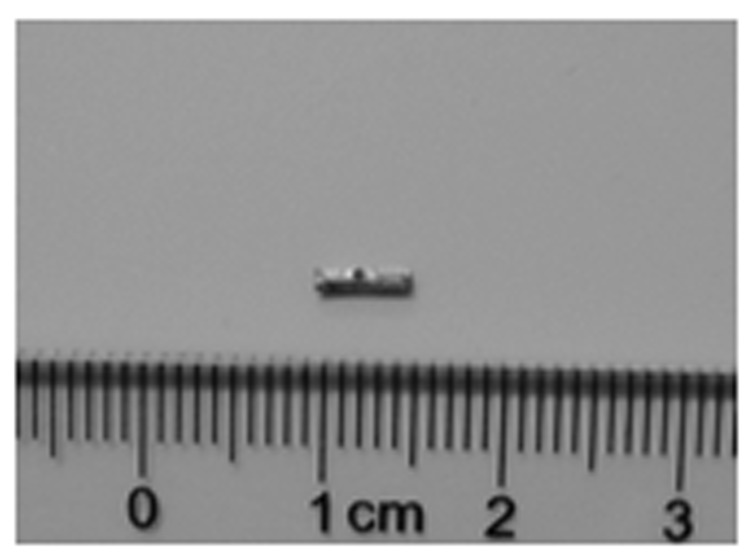
A biodegradable biliary stent made of Mg-6Zn alloy, 1 mm in diameter and 5 mm in length. The stent is U-shaped and has a small hole drilled in it.

**Figure 2.**
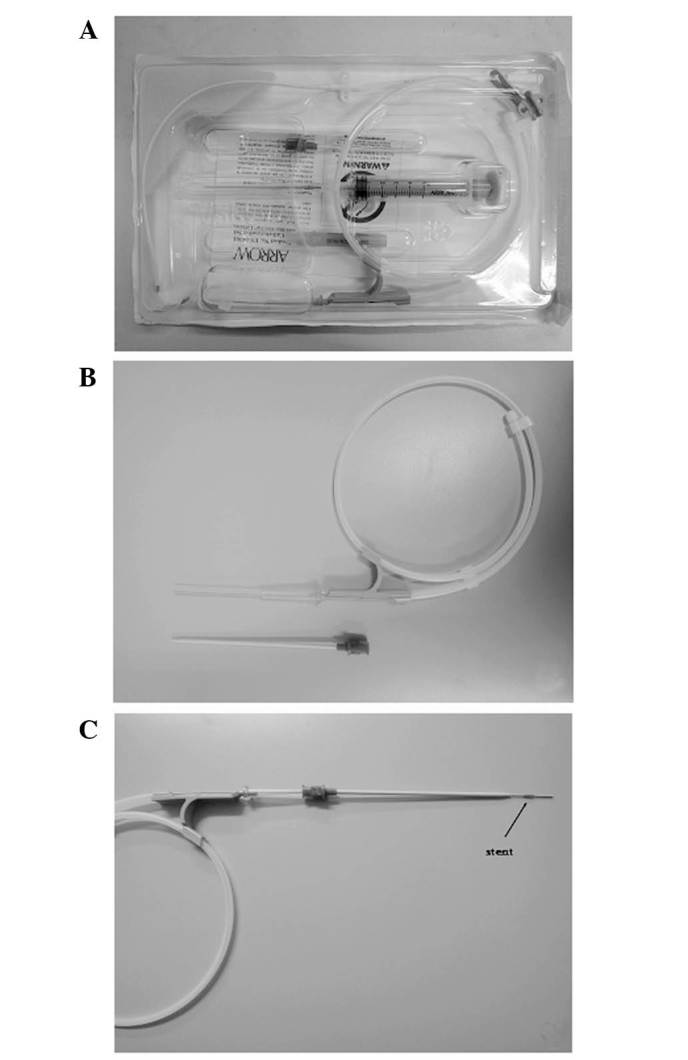
A central venous catheterization set (REF CS-24301-E; Arrow International, Inc.) was used as a special stent introducer system. (A) The central venous catheterization set; (B) a plastic jacket tube and a metal guide wire. (C) The stent was mounted onto the stent introducer system.

The biodegradable stents made of Mg-6Zn alloy, donated by Shanghai Origin Material and Medical Technology Co. Ltd. (Shanghai, China), were made with high purity Mg (99.99%) and high purity zinc (Zn; 99.999%) under a clean process. The chemical composition of the Mg-6Zn alloy is shown in Table I. Materials were prepared as previously described (13). The biodegradable biliary stents were U-shaped and possessed a luminal diameter of 1.0 mm and a length of 5 mm (Fig. 1). A small hole was drilled in the middle of the stent to enable the stent to be sutured to the CBD wall. The system used in endoscopic retrograde cholangiopancreatography (ERCP) prompted the use a central venous catheterization set (REF CS-24301-E; Arrow International, Inc., Reading, PA, USA) as a special stent introducer system (Fig. 2). The parts used included a plastic jacket tube and a metal guide wire, which formed the CBD introducer system.

### Animal model and study design

The animal experiment was conducted according to the Guidance Suggestions for the Care and Use of Laboratory Animals (issued by the Ministry of Science and Technology of the People’s Republic of China), and was approved by the Ethics Committee of the Sixth People’s Hospital of Shanghai Jiao Tong University (Shanghai, China). The animals were supplied by the Sino-British Sippr/BK Lab Animal Ltd., Co. [license no. SCXK(hu)2008-0016; Shanghai, China]. Twelve adult New Zealand rabbits with a mean body weight of 2.5±0.5 kg were used in the experiments.

**Figure 3.**
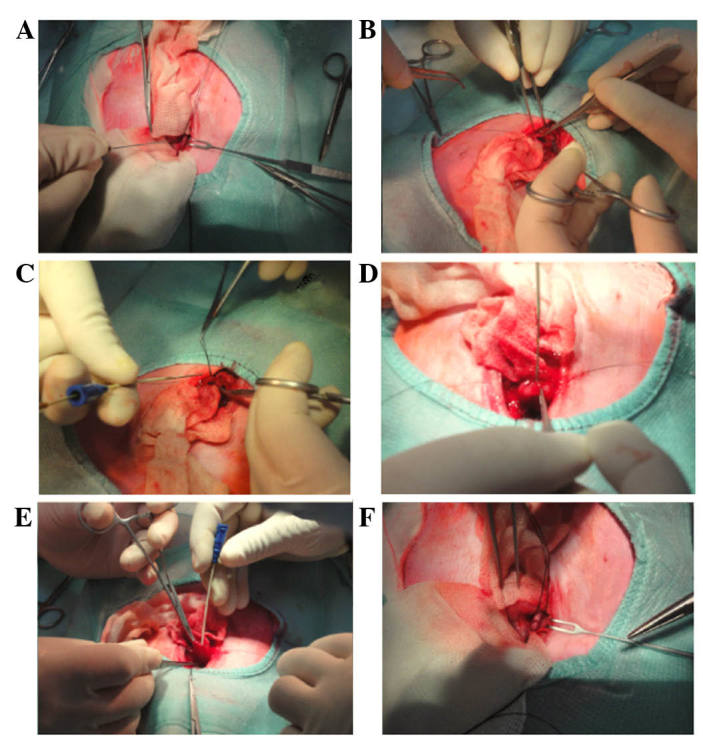
Process for placing an advanced stent into the rabbit common bile duct (CBD). (A) The CBD was identified and ligatured as a marker; (B) a 15-mm longitudinal incision was cut into the duodenum, 5 mm away from the duodenal papilla; (C) the stent introducer system was assembled and prepared to place the stent; (D) a metal guide wire was placed into the CBD, the plastic jacket tube was pushed down and a stent was inserted into the CBD; (E) the guide wire and plastic jacket tube were gradually withdrawn; (F) the stent was sutured to the CBD inner wall.

Rabbits were placed under general anesthesia by the intravenous administration of sodium pentobarbital at a dose of 30 mg/kg body weight. All surgical procedures were carried out under sterile conditions. Through a midline laparotomy, the CBD was exposed and dissected along the CBD to 10 mm from the duodenal wall. The mean diameter of the CBD was 1.5 mm and the mean length was 12 mm in 10 rabbits. A ligature was loosely placed on the CBD as a marker of the location of desired stent placement. A 15-mm longitudinal incision was made in the duodenum, 5 mm away from the duodenal papilla. The stents were mounted onto the special stent introducer system. When the metal guide wire arrived at the ligature marker on the CBD, the plastic jacket tube was pushed back. Then, the stent was advanced along the duodenal papilla and the metal guide wire was withdrawn. As the CBDs of rabbits are translucent, it was possible to view the stent in the CBD under direct vision and to suture the stent to the CBD wall through the small hole on the stent. The plastic jacket tube was then withdrawn, and the duodenum and abdomen was closed (Fig. 3).

Computed tomography (CT) scanning of the CBD stent *in vivo* was conducted after the surgical procedures immediately and postoperatively for 1–3 weeks. To investigate changes in the pancreatic function of the rabbits, the serum lipase (LPS) values of each rabbit were determined. In brief, a 1-ml blood sample was collected prior to the surgical procedures and postoperatively for 1–3 weeks from the ear vein of the rabbit using a vein puncture into a heparinized syringe. The samples were then immediately analyzed using an automatic blood biochemistry analyzer (Hitachi 7600-020; Hitachi High-Technologies, Tokyo, Japan; LPS kit was provided by Koch Industries, Inc., Wichita, KS, USA). Eleven rabbits were sacrificed at 3 weeks after surgery.

### Statistical analysis

Statistical analysis was performed with SPSS 18.0 software package (SPSS Inc., Chicago, IL, USA). The experimental values were analyzed using the paired-samples t-test and expressed as the mean values ± standard deviation (SD). One-way ANOVA analysis was calculated to determine differences between groups for each evaluated parameter at each time point. Non-parametric tests [κ independent samples tests (Kruskal-Wallis Test)] were calculated when equal variances were not assumed in one-way ANOVA. P<0.05 was considered to indicate a statistically significant result.

## Results

Twelve rabbits underwent CBD stent insertion using the stent introducer system. One animal died due to an anesthetic accident. The remaining 11 rabbits that were included in the final analysis grew well and their activities, diet and drinking were normal. When these 11 rabbits were sacrificed after 3 weeks, no jaundice or bile leakage was observed.

**Figure 4.**
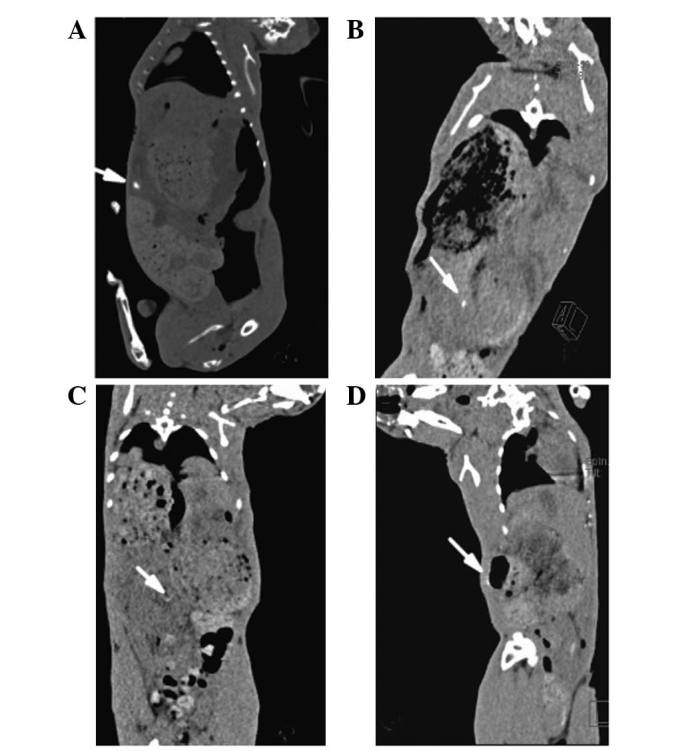
Computed tomography scan on (A) the day of surgery and (B) the 1st, (C) 2nd and (D) 3rd week post-implantation. The stents degraded gradually.

**Figure 5.**
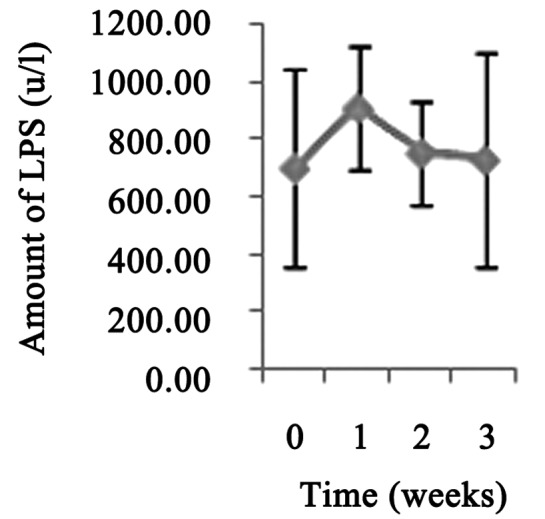
Serum lipase (LPS) levels of the rabbits prior to and following surgery.

CT scanning for these 11 rabbits suggested that the biodegradable Mg-6Zn stent was successfully placed into the CBD. Since the stents degraded gradually, after 3 weeks, the stent was rarely identified (Fig. 4). Although the levels of LPS 1 week postoperatively were slightly higher than the preoperative levels, no statistically significant differences were observed (P>0.05; Fig. 5).

## Discussion

Following CBD exploration, it is important to insert a T-tube in the CBD to provide a support and to maintain an open CBD (14). However, T-tube drainage is controversial since it appears to prolong the hospital stay and is associated with an increased cost of care (15). There are risks for T-tube placement, including increased morbidity or mortality secondary to biliary infection, migration of the tube causing bile duct obstruction, or bile leaks or peritonitis following the removal of the tube (1,16,17). Therefore, there is a great clinical requirement for biodegradable CBD stents. Animal experiments for new surgical biliary stents made of novel materials are being conducted more frequently than ever before. However, in practice, it is difficult to perform a safe choledochotomy for small animals, such as rabbits.

In the present study, a biodegradable Mg-6Zn alloy CBD stent was inserted into the CBDs of rabbits via a central venous catheterization set. At present, there are several materials that may be used to create a biodegradable stent and in which it is easy to drill a hole in the surface. Since the CBD of rabbits is translucent, it is possible to observe these stents in the CBD, and the hole in the middle of the stent under direct vision. The stent was fixed to the CBD wall by a suture through the hole. Due to the stent compression of the sutured wall of CBD, no bile leakage was observed following suture completion. Fixing the stent to the CBD wall has certain advantages. Firstly, it demonstrates whether a stent may induce histological changes where it contacts with the CBD. Secondly, a fixed CBD stent is required for study of further stent biodegradation behavior .

One main problem to be overcome is the requirement that the progression of the stent through the duodenal papilla should be completed within a short time, which may reduce the adverse effects on the duodenal papilla. In order to clearly explore the duodenal papilla and the relationship between the papilla and the direction of the CBD, a relatively large longitudinal incision (15 mm) was cut in the duodenum, 5 mm away from the duodenal papilla. The results of serum LPS assays showed that this method for CBD stent placement had no significant impact on the function of the pancreas.

In conclusion, although the advancement of the stent through the duodenal papilla produces extra risks, our methods appear to be feasible and safe for the placement of stents into the CBDs of small animals. This study increases the number of animals available for CBD studies and may improve the quality of experiments concerning CBD stents in animals.

## Figures and Tables

**Table I.** Chemical composition of Mg-6Zn alloy.

Material	Chemical composition (weight %)							
	Fe	Si	Ni	Cu	Al	Mn	Zn	Mg
Mg-6Zn	0.0038	0.0016	0.0005	0.0005	0.0085	0.0004	5.6210	94.3637

## Abbreviations:

CBD: common bile duct
